# Nanogel: A Versatile Nano-Delivery System for Biomedical Applications

**DOI:** 10.3390/pharmaceutics12030290

**Published:** 2020-03-23

**Authors:** Yanlong Yin, Ben Hu, Xiao Yuan, Li Cai, Huile Gao, Qian Yang

**Affiliations:** 1 Collaborative Innovation Center of Sichuan for Elderly Care and Health, No. 783, Xindu Avenue, Xindu District, Chengdu 610500, Sichuan, China; rain1505496802@163.com (Y.Y.); huben826@gmail.com (B.H.); 2 School of Pharmacy, Sichuan Province College Key Laboratory of Structure-Specific Small Molecule Drugs, Chengdu Medical College, No. 783, Xindu Avenue, Xindu District, Chengdu 610500, Sichuan, China; yuanxiao0617@163.com (X.Y.); caili0000913@163.com (L.C.); 3 Key Laboratory of Drug-Targeting and Drug Delivery System of the Education Ministry, Sichuan Engineering Laboratory for Plant-Sourced Drug and Sichuan Research, Center for Drug Precision Industrial Technology, West China School of Pharmacy, Sichuan University, Chengdu 610064, China

**Keywords:** multifunctional nanogels, crosslinking, stimuli-responsive, delivery, combinational therapy

## Abstract

Nanogel-based nanoplatforms have become a tremendously promising system of drug delivery. Nanogels constructed by chemical crosslinking or physical self-assembly exhibit the ability to encapsulate hydrophilic or hydrophobic therapeutics, including but not limited to small-molecule compounds and proteins, DNA/RNA sequences, and even ultrasmall nanoparticles, within their 3D polymer network. The nanosized nature of the carriers endows them with a specific surface area and inner space, increasing the stability of loaded drugs and prolonging their circulation time. Reactions or the cleavage of chemical bonds in the structure of drug-loaded nanogels have been shown to trigger the controlled or sustained drug release. Through the design of specific chemical structures and different methods of production, nanogels can realize diverse responsiveness (temperature-sensitive, pH-sensitive and redox-sensitive), and enable the stimuli-responsive release of drugs in the microenvironments of various diseases. To improve therapeutic outcomes and increase the precision of therapy, nanogels can be modified by specific ligands to achieve active targeting and enhance the drug accumulation in disease sites. Moreover, the biomembrane-camouflaged nanogels exhibit additional intelligent targeted delivery features. Consequently, the targeted delivery of therapeutic agents, as well as the combinational therapy strategy, result in the improved efficacy of disease treatments, though the introduction of a multifunctional nanogel-based drug delivery system.

## 1. Introduction

With the development of advanced nanotechnology in recent decades, nanocarriers have emerged and gained popularity in biomedicine [1]. Nanocarriers serve not only as carriers of routine chemotherapeutic agents due to their drug encapsulation capacity but also as platforms for combinational therapy, multifunctional diagnosis and theranostics. As an ideal multifunctional drug delivery system (DDS), nanocarriers have been applied for various disease therapies, such as passive targeting due to the enhanced permeability and retention (EPR) effect, active targeting facilitated by ligand modification of the surface of nanoplatforms, and site-specific and time-controlled drug delivery strategies mediated by stimuli-responsive materials [2].

Nanogels, a type of systemic drug delivery carrier, are hydrogels with a three-dimensional (3D) tunable porous structure and a particle size in the submicrometer range, from 20 to 250 nm; nanogels can be discriminated from microgels, which have a particle size ranging from 1 to 350 µm, and in situ-forming hydrogels, which facilitate local delivery [3]. Nanogels are composed of various natural polymers, synthetic polymers, or combinations thereof, which contributes to the encapsulation of small molecules, oligonucleotides, and even proteins. These unique properties equip nanogels with the abilities to enable drug delivery, diagnostics, and imaging [4].

As a kind of hydrogel, nanogels retain the highly hydrated nature and shrinking-swelling properties of hydrogels under different environmental conditions [5]. Their 3D structure enables the encapsulation of hydrophobic or hydrophilic drugs in their internal network, potentially protecting these drugs from degradation during storage or in circulation (such as degradation due to hydrolysis or enzymolysis) [6]. Unlike typical nanoparticles (NPs), nanogels exhibit a tunable particle size, particle shape, and sensitivity to pH, temperature, ionic strength, redox conditions and other external stimuli, giving them effective controlled drug release properties [7]. Furthermore, nanogels can be tailored to be multifunctional and targeted, and their circulation time can be prolonged by surface modification [8]. Based on the above advantages, nanogel-based DDSs have become popular and had significant impacts in recent years.

**Scheme 1:**
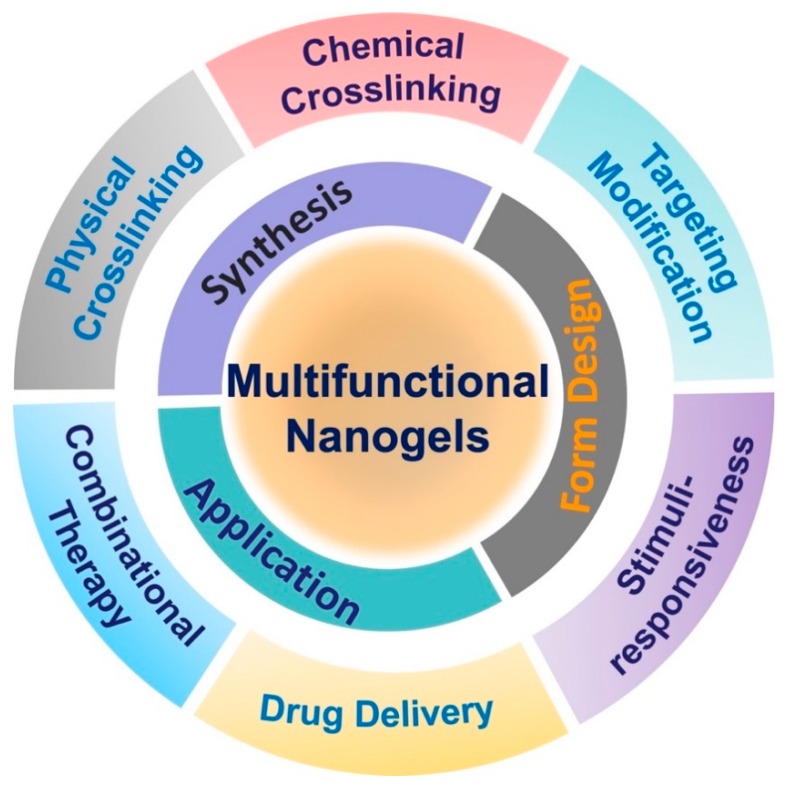
Schematic illustration of multifunctional nanogels and their biomedical application in drug delivery.

In this review, we summarize recent developments in nanogels and their biomedical application in drug delivery (Scheme 1). In addition to the composition of nanogels and methods of their preparation, the construction of various pH-responsive, temperature-responsive, dual pH/temperature-responsive and triple pH/temperature/redox-responsive nanogels are presented in detail. Moreover, targeted ligands, such as promising small molecules, peptides and antibodies on the surface of nanogels, enable the development of nanogels with active targeting properties. Finally, the biomedical applications of nanogels are discussed in detail.

## 2. Construction of Nanogels

Based on the different structures and building blocks in nanogels, the methods of nanogel synthesis can be divided into chemical crosslinking and physical self-assembly. The nanogel formed by chemical crosslinking exhibited preferable stability compared to the physical crosslinking through the covalent crosslinking between functional groups on polymer chains. Meanwhile, the reversible connections of physically crosslinked nanogels are commonly dependent on the noncovalent interactions, which mainly include hydrogen bonding, Van der Waals force, hydrophobic interaction, host-guest interaction, electrostatic interaction, and so on [9]. Although the interaction of physical noncovalent bonds is relatively weaker than that of chemical covalent crosslinking, the process of physical self-assembly is more flexible and convenient, because it does not require complex reactions [10,11]. This section discusses the synthetic methods and examples of nanogel preparation are discussed.

### 2.1. Physical Crosslinking

Physically crosslinked nanogels are supramolecular particles consisting of polymer molecules formed through noncovalent interactions. The sizes of nanogels can be influenced by the polymer concentration and different environmental conditions, such as ionic strength, temperature, and pH, during nanogel preparation.

**Figure 1:**
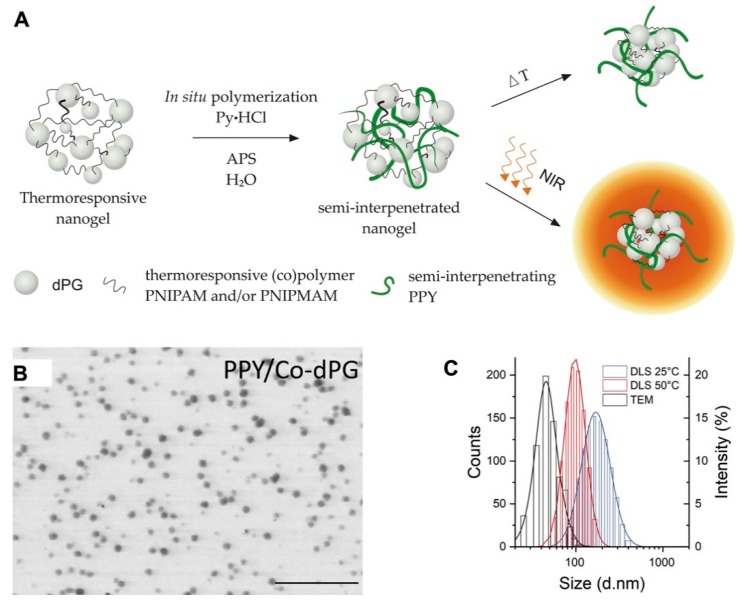
(**A**) The scheme for synthesis of dual responsive nanogels via semi-interpenetration method. The N-isopropylacrylamide (NIPAm)-based polymer network and interpenetrated polypyrrole (PPY) enabled the nanogels with thermoresponsive property and near-infrared (NIR)-induced photothermal convention, respectively; (Pyrrole∙HCl (Py∙HCI) as the base and ammonium persulfate (APS) as an initiator were introduced for polymerization); the TEM images (**B**) and hydrodynamic size distribution (**C**) of PPY/Co-dPG nanogels. Adapted from (Journal of Controlled Release. 2019, 311–312, 147–161). Copyright (2020) with permission from Elsevier Science.

The semi-interpenetration method was described as physically incorporating an insoluble molecule into a crosslinked polymer network, and the obtained nanogels can then extend the new properties of the incorporated molecule. Polypyrrole (PPY) with photothermal convention, which could be used for photoacoustic (PA) imaging, was introduced into dendritic polyglycerol (dPG) cross-linked poly(*N*-isopropylacrylamide-*co*-*N*-isopropylmethacrylamide) nanogels (p(NIPAm-*co*-NIPMAm)) by semi-interpenetration to form the PPY/Co-dPG nanogels. PPY/Co-dPG nanogels with near infrared (NIR)-responsive and hermoresponsive properties could be used for PA imaging-guided photo thermotherapy with NIR irradiation (Figure 1) [12].

### 2.2. Chemical Crosslinking

Beyond physical crosslinking, chemical crosslinking is the most developed and more versatile strategy for nanogel construction. Chemical crosslinking methods include polymerization by emulsion, reversible addition-fragmentation chain transfer (RAFT), click chemistry crosslinking, and photo-induced crosslinking [13]. Amino crosslinking is often used for the preparation of biodegradable nanogels based on amino acids [14].

#### 2.2.1. Inverse Emulsion Polymerization

Inverse emulsion polymerization is a polymerization reaction initiated by the continuous emulsification of water-in-oil emulsifiers in the oil phase. The sizes of nanogels can be regulated by many factors, such as the surfactant, feed ratio of the monomer and crosslinker, and pH [11]. For example, Ashrafizadeh et al. synthesized zwitterionic poly(AA-BA-EGDMA) nanogels by using ethylene glycol dimethacrylate (EGDMA), butyl acrylate (BA) and acrylic acid (AA) as monomers [9]. Using *N*,*N*′-methylenebis(acrylamide) (BIS) and *N*-acryloyl-l-glutamic acid (l-AGA), Peres and co-workers prepared poly(l-AGA) and poly(l-AGA-*co*-BIS) hydrogels by inverse emulsion polymerization, which demonstrated that the degree of hydrogel swelling increased with variation of pH [15]. Furthermore, the presence of carboxylic acid and amide groups in a polymer network plays important roles in the physicochemical properties of the polymer network, thereby affecting its hydrophilicity and hydration.

#### 2.2.2. Reversible Addition-Fragmentation Chain Transfer (RAFT) Polymerization

Through RAFT, a polymer undergoes a series of reactions with dithioester compounds; these reactions include reversible addition, reversible degradation of adducts, and chain transfer reactions and control the molecular weight of the polymer during free radical polymerization. RAFT technology can change the micelle structure of amphiphilic polymers by altering the length, configuration and properties of the polymers. Poly(*N*-vinylcaprolactam) (PVCL) has become an interesting biocompatible and temperature-sensitive polymer [16]. In addition, poly(vinyl acetate) (PVAC) is an attractive polymer that can be used to adjust the phase transition temperature of PVCL, which is known as a kind of biocompatible and temperature-sensitive polymer, by promoting the noncovalent hydrophobic interactions between VAC and VCL. By adjusting the ratio of VAC and VAL during RAFT polymerization, Etchenausia et al. obtained a series of thermoresponsive polymers which they named as PEG-b-P(VAC-*co*-VCL), with xanthate-terminated poly(ethylene glycol) (PEG-X) as a polymeric chain transfer agent [17].

#### 2.2.3. Click Chemistry Crosslinking Polymerization

**Figure 2:**
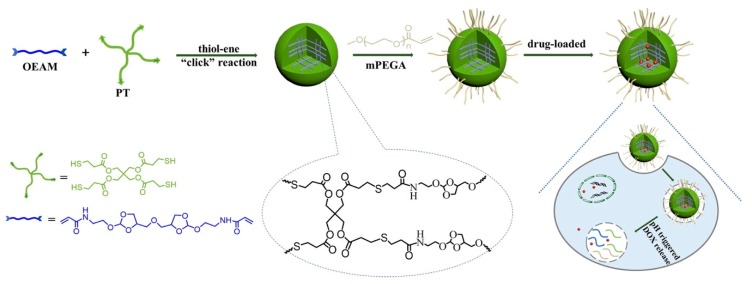
The scheme for synthesis of pH-sensitive nanogels via thiol-ene“click” reaction, and the mechanism for drug loading and intracellular drug release. Adapted from (Journal of Colloid and Interface Science. 2017, 508, 282–290). Copyright (2020) with permission from Elsevier Science.

In recent decades, emergent hydrogels and nanogels have been associated with click chemistry due to its high reactivity, high yield and superb selectivity [18]. Click chemistry, which includes the copper(I)-catalyzed azide-alkyne (CuAAC) click reaction, the copper-free click reaction, and pseudo-click chemistry, has become the most promising strategy for the preparation of nanogels. For instance, a pH-responsive nanogel was obtained through thiol-ene click chemistry, an efficient method of nanogel preparation without byproducts, by polymerization with methoxy polyethylene glycol acrylate (mPEGA), pentaerythritol tetra(3-mercaptopropionate) (PT) and ortho ester diacrylamide (OEAM) [19]. The network of the obtained nanogels was introduced to capture the molecule doxorubicin (DOX), and acid-labile monomer OEAM enabled the DOX-loaded nanogel displayed a pH-triggered release profile with the intracellular acidic environment (Figure 2).

#### 2.2.4. Photo-Induced Crosslinking Polymerization

The application of irradiation for the preparation of nanogels is becoming popular due to its bacteriostatic effect, additive-free nature, multifunctional nature, tunable particle diameter and ability to promote crosslinking. In the process of irradiation, water molecules break down into hydroxyl radicals and hydrogen atoms with the potential to convert polymers into microradicals, leading to intermolecular crosslinking, which promotes nanogel formulation [20]. Consequently, the crosslinking density can be adjusted by regulating the wavelength or energy of the laser [21]. In addition, there are optical crosslinking methods using photoinitiators; for instance, Irgacure 2959 was applied to prepare lipid-coated nanogels which could reduce the drug release rate of docetaxel, compared to that of uncrosslinked drugs [22,23].

## 3. Stimuli-Responsive Nanogels

The use of stimuli-responsive systems for drug delivery has received increasing attention and mainly utilizes the special conditions of the tumor microenvironment, including a low pH, high temperature, and high glutathione (GSH) concentration, to achieve targeted drug delivery and release [24]. In this way, the use of stimuli-responsive systems not only decreases the drug dose and systemic toxicity but also weakens toxicity to normal tissues [25]. Nanogels have been widely used as stimuli-responsive systems due to their stability, ease of synthesis, high drug-loading capacity, and ability to be modified in multiple ways. Importantly, nanogels are more highly responsive to mutative environments than other DDSs due to their unique 3D network structures, which easily change in different environments to control drug release [26]; therefore, several common stimuli-responsive nanogels are introduced below.

### 3.1. Thermo-Responsive Nanogels

Thermo-responsive nanogels are among the most attractive intelligently responsive DDSs. Thermoresponsive nanogels present shrinkage-swelling behavior with variation in the environmental temperature, which enables a controlled release rate of the drug loaded inside. Moreover, accumulation in the disease-related microenvironment and the improved intracellular uptake efficiency could be achieved by stimulated reduction in particle size, and further benefit the therapeutic outcomes [27,28,29].

**Figure 3:**
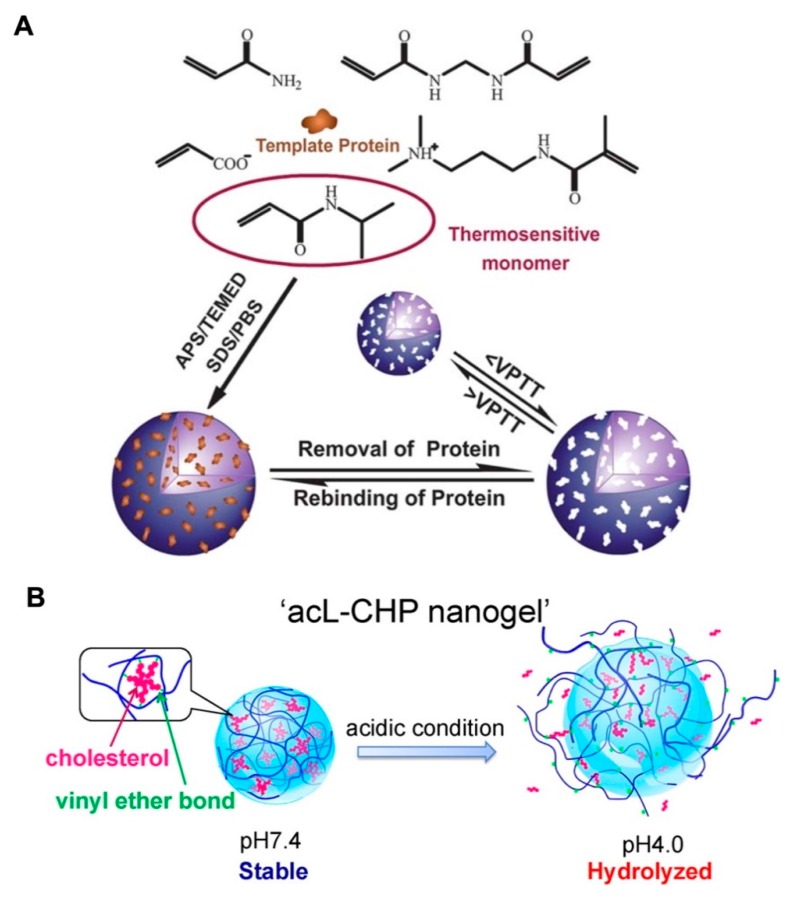
(**A**) Schematic illustration of the aqueous imprinting method for protein-loaded thermoresponsive nanogels (with APS as an initiator and sodium dodecyl sulfate (SDS) as an emulsifier). The change in temperature below or above VPTT enabled the removal or rebinding of protein. Republished with permission of Royal Society of Chemistry, from [Soft Matter, 2013, 9, 3840–3850. Edition Number 38, Copyright (2013)]; permission conveyed through Copyright Clearance Center, Inc. (**B**) By hydrolysis of cholesteryl vinyl ether bond at pH 4.0, the acid-labile cholesteryl-bearing pullulan nanogels (acL-CHP) resulted in swelling-collapse and presented the acid-triggered drug release. Adapted with permission from (Biomacromolecules 2013, 14, 56-63). Copyright (2013) American Chemical Society.

NIPAm is a typical thermosensitive derivative that has been widely applied in biomedicine [30,31,32]. Below 32 °C (the lower critical solution temperature (LCST) of NIPAm), NIPAm is hydrophilic. It becomes hydrophobic with the agglomeration of NIPAm units and causes the system to shrink when the temperature exceeds its LCST. Moreover, the LCST of pNIPAm can be regulated by chain modifications. Hence, pNIPAm-based copolymers with thermosensitive properties were synthesized by chemical crosslinking with other monomers to form various DDSs, including nanogel [33,34], and hydrogel [35,36,37]. In the recent report, by initiating the polymerization of NIPAm with AA and N-[3-(dimethylamino) propyl] methacrylamide (DMAPMA), the volume phase transition temperature (VPTT) of pNIPAm-based nanogels was adjusted to near the physiological temperature (37 °C). with APS as an inissiaer and sodium dodecyl sulfate (SDS) as an emulsifier. This thermo-sensitive nanogel was introduced for protein loading by aqueous imprinting precipitation. More importantly, the template protein lysozyme exhibited imprint-release property accompanying with the temperature-dependent shrinking-swelling of nanogel, indicating the promising controlled delivery ability (Figure 3A) [38].

### 3.2. pH-Responsive Nanogels

The pH-dependent swelling-shrinking behavior of the nanogel system is mainly attributable to the ionizing groups, which could deform by ionization or deionization in response to variation in the pH value. Some scientific research has reported that the microenvironments of tumor tissues (pH 6.5–7.2) and tumor cells (pH 4.5–5.0 in lysosomes, and pH 5.0–6.5 in endosomes) are acidic, when compared with the physiological pH of 7.4 in the blood circulation and normal tissues [39,40,41]. The pH-sensitive monomers methacrylic acid (MAA) and methyl ester (MA) were reported to polymerize nanogels, which maintained a swollen state at basic pH conditions with high permeability. Accompanied by decreasing pH values, the deionization of MAA and MA would lead to the shrinkage of nanogels, resulting in the entrapment of the hydrophobic fluorescent indicator oligothiophene (TF) and the hydrophilic drug DOX. Moreover, the enhanced release of DOX was observed at pH 5.5, which could be attributed to the further shrinkage of pH-sensitive nanogels and the protonation of DOX [42]. Besides the synthetic polymers, the natural polysaccharide–based nanogels with shrinkage-collapse variation were designed for precisely controlled drug release with the stimulus of disease microenvironment. Cholesteryl-modified pullulan polymers were reported to self-assemble under physiological conditions to form a stable network structure of acid-labile cholesterol-bearing pullulan (acL-CHP). However, the acid-catalyzed hydrolysis of cholesteryl vinyl ether bonds occurred at pH 4.0, leading to the swelling of the acL-CHP nanogels, and finally resulting in collapse of the nanogels. The acL-CHP was expected to be a promising carrier for protein delivery with controlled release (Figure 3B).

### 3.3. Magnetic-Responsive Nanogels

Besides the magnetic-targeting under extra magnetic field, the magnetic nanoparticles (MNPs) can realize hyperthermia under the conditions of alternative magnetic field (AMF) [43,44]. Hence, MNPs and the temperature-sensitive nanogels were applied to construct the hybrid nanogels and loaded with chemical drug DOX (DOX-MagNanoGels). Nanogels provide opportunities to co-encapsulate MNPs and chemical drugs due to the 3D network structure. Moreover, the shrinking-swelling property of nanogels guarantees stimuli-drug release when AMF is applied. The DOX-MagNanoGels not only displayed enhanced internalization of cancer cells and release of DOX because of the shrinkage of nanogels by MNPs-induced magnetic hyperthermia, but also possess the possibility for magnetic resonance imaging (MRI) and magnetic targeting in cancer diagnosis and therapy [45].

### 3.4. Ultrasound-Responsive Nanogels

Ultrasound (US)-mediated drug delivery systems are widely used in transdermal administration and central nervous system (CNS) disease treatment [46,47]. Moreover, a US-responsive delivery system was also introduced for anticancer therapy based on the advantages for deep penetration of acoustic wave. When US was applied, the liquid US agent perfluorohexane (PFH) evaporated to gas, benefitting the triggered drug release [48]. A redox-sensitive hybrid nanogel (MSNss-gel) was synthesized by the mesoporous silica nanoparticles’ (MSNs) core and polymer shell, and was utilized for hydrophilic drug DOX and hydrophobic PFH co-loading. The collapse of the polymer shell in the microenvironment of the tumor region enabled the controlled DOX release and also improved the US contrast imaging in cancer therapy [49].

### 3.5. Multistimuli-Responsive Nanogels

**Figure 4:**
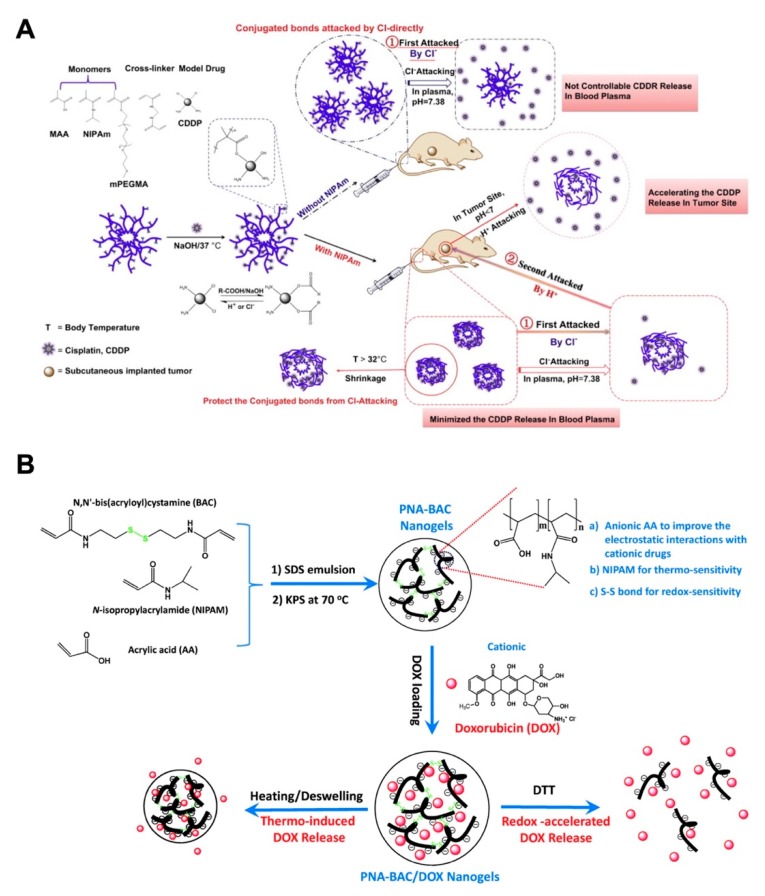
(**A**) The synthesis of temperature and pH duel-responsive nanogel, and the drug release mechanism of cisplatin (CDDP)-conjugated nanogel when at circulation and in tumor region. Adapted with permission from (Biomaterials. 2013, 34, 8726–8740). Copyright (2013) American Chemical Society. (**B**) The in situ polymerization of thermo/redox-sensitive nanogels (with potassium persulfate (KPS) as an initiator and SDS as an emulsifier), and the doxorubicin (DOX)-loaded nanogels exhibited both thermo and redox triggered drug responsive release. Republished with permission of Royal Society of Chemistry, from [Journal of materials chemistry. B, Materials for biology and medicine, 2015, 3, 4221–4230. Edition Number 57, Copyright (2015)]; permission conveyed through Copyright Clearance Center, Inc.

Monoresponsive nanogels cannot maintain the controlled drug release effectively, so nanogels with dual- or multi-stimuli responsiveness have attracted widespread attention [50,51,52,53]. Among the multiresponsiveness combinations, pH–temperature dual-sensitivity combinations have been studied with considerable advances. Peng and coworkers innovated a kind of dual-stimuli nanogel polymerized from NIPAm, MAA and PEGMA that responded to temperature and pH values. The cross-linked p(NIPAm-MAA-PEGMA) nanogels displayed the excellent drug loading and release behavior for hydrophilic model drugs with different molecular weights [54]. Furthermore, the p(NIPAm-MAA-PEGMA) nanogels were introduced as the pH-thermal dual responsive carriers for loading cisplatin (CDDP), which was conjugated with the COOH group from MAA, and the release could be triggered by H^+^ and Cl^−^ attack. The thermoresponsive nanogels could reduce the Cl^−^-triggered drug release during circulation, while the acidic environment in the tumor region would achieve the controlled release of CDDP, improving antitumor efficacy and alleviating adverse effects (Figure 4A) [55]. Besides the physical condition, the intracellular responsive release of glutathione (GSH), an essential antioxidant that specifically reduces the disulfide bonds, was reported at a relatively high concentration in cancer cells (approximately 2–10 mM), which is approximately 1000 times the value of approximately 2–20 μM in normal cells [56]. The monomers NIPAM and AA were crosslinked by *N*,*N*’-bis(acryloyl)cystamine (BAC) to prepare the PNA-BAC nanogels with thermo, pH-, and redox-sensitivity for DOX delivery [57]. In this research, triple-responsive PNA-BAC-DOX nanogels displayed the accelerated DOX release in the presence of 4 mM DTT, which mimicked the tumor cell environment. Meanwhile, the acidic conditions were beneficial for the DOX release from PNA-BAC-DOX nanogels, which was in accordance with the tumor microenvironment (Figure 4B).

### 3.6. Modification of Nanogels for Active Targeting

**Figure 5:**
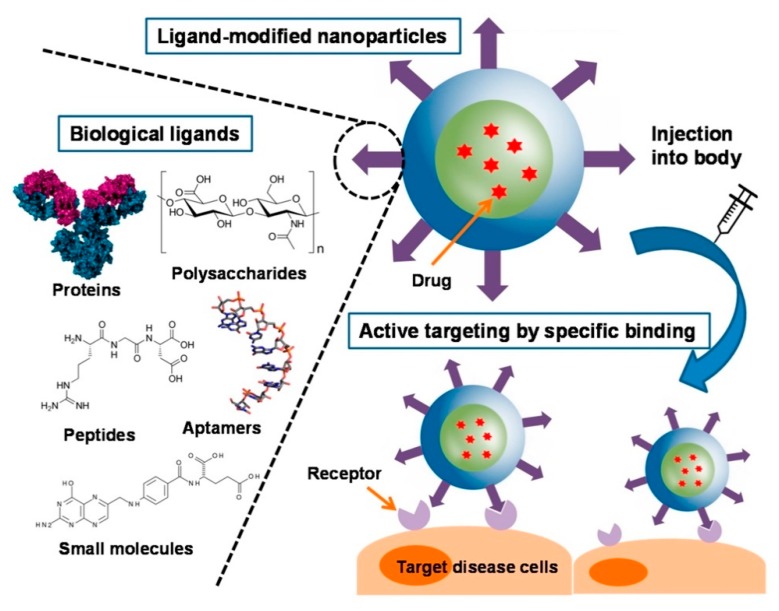
Schematic illustration of biological ligands used for active targeting in nanoparticles. Adapted from (Cancers. 2019, 11, E640. Multidisciplinary Digital Publishing Institute).

Like other types of NP, nanogels can be provided not only passive targeting ability by adjustments to their size, shape or surface property, but also active targeting by their surface modification, further improving the accumulation of drugs in disease region [58]. Active targeting can be achieved through the interaction of ligands and specific cellular or subcellular receptors. Moreover, the biological ligands such as small molecules, proteins, peptides, polysaccharides were employed for the surface modification of NPs (Figure 5) [59].

#### 3.6.1. Small-Molecule Conjugation

In recent studies of antitumor targeted therapies, folic acid (FA) has become a prominent target that can specifically interact with cells overexpressing folate receptors (FRs). FRs are overexpressed in human tumor tissues, especially in ovarian cancer tissues, but are seldom expressed in normal tissues. The differential expression of FR in ovarian cancer and other tumor tissues makes it an attractive biomarker for the diagnosis and treatment of tumors [60]. The block copolymer poly (ethylene oxide)-*b*-poly(methacrylic acid) (PEO-*b*-PMA) was loaded with CDDP and DOX, and further modified with FA to obtain pH-sensitive nanogels. The intracellular uptake results show that the uptake rate of FA nanogels in FA-positive cells (approximately 80%) was much higher than that in FA-negative cells (approximately 20%). In vivo data showed that CDDP-loaded FA-nanogel treatment resulted in an obvious therapeutic effect in ovarian cancer-bearing animal models with reduced toxicity to the kidney [61].

#### 3.6.2. Peptide Conjugation

**Figure 6:**
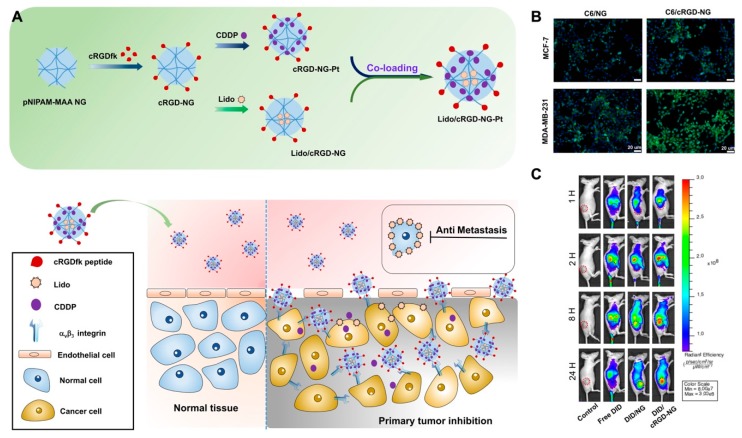
(**A**) The scheme for the preparation of Lido/cRGD-NG-Pt nanogels and internalization process of cRGD modified nanogel in the tumor region; (**B**) in vitro confocal fluorescence images illustrated that the cRGD modified nanogel present enhanced cell uptake efficacy for αVβ3 integrin-positive MDA-MB-231 tumor cells; (**C**) in vivo fluorescence imaging of MDA-MB-231 tumor model after treatments with fluorescent probe-labeled nanogels (DID/cRGD-NG). MR Imaging demonstrated the tumor targeting property of MRI probe-labeled nanogels (Fe_3_O_4_/cRGD-NG). Adapted with permission from (ACS Appl. Mater. Interfaces 2018, 10, 25228−25240). Copyright (2018) American Chemical Society.

Recently, the active targeting with some peptide ligands has been extensively investigated in both therapy and imaging. For instance, the tumor-homing peptide LyP-1, which was reported to bind preciously with p32 protein of a series of tumor cells, could be conjugated with nanoparticles to realize enhanced tumor targeting. Furthermore, improved therapeutic outcomes resulting from treatment with these modified nanoparticles were achieved by photo-thermotherapy, photo-chemotherapy and photo-immunotherapy in our previous work [62,63,64]. In addition to LyP-1, the RGD peptide, a tripeptide consisting of L-arginine, glycine and L-aspartic acid, is a cell adhesion sequence that mimics cell adhesion proteins and can bind to integrin receptors on angiogenic endothelial cells and tumor cells, such as malignant glioblastoma cells, bladder cancer cells and α_V_β_3_ integrin receptor-overexpressing breast cancer cells [65]. Moreover, the cyclic RGD (cRGD) peptide has also been demonstrated to maintain the affinity to tumor cells. Hence, the cRGD-modified nanogels could be the versatile and promising drug carriers for tumor-targeting strategies. In our previous study, acid-sensitive pNIPAM-*co*-MAA nanogels (NG) were conjugated with cRGD peptide. Meanwhile, CDDP and lidocaine (Lido) were co-loaded by the active targeting nanogel system (Lido/cRGD-NG-Pt). The results of in vivo fluorescence imaging and magnetic resonance (MR) imaging demonstrate that the ligand-modified nanogels exhibited enhanced tumor region targeting and accumulation, which could be attributed to the specific internalization of the cRGD peptide. Consequently, by combining the tumor region enrichment with controlled drug release in the tumor environment, the dual drug-loaded nanogels displayed enhanced therapeutic outcomes in MBA-MD-231 breast cancer model (Figure 6) [66].

#### 3.6.3. Antibody Conjugation

Compared with specific receptors on the surface of tumor cells, antibody-modified nanogels show a higher affinity for binding sites, allowing them to achieve higher targeting and precision, so antibodies are widely used as ligands in the modification of nanogels [67,68]. In addition, antibodies can be applied not only as the ligands but also as therapeutic agents in cancer treatment; for instance, antibody-dependent cell-mediated cytotoxicity (ADCC) was indicated to inhibit cellular signaling pathways related to tumor growth and initiation [69,70]. In a previous report, an anti-STn antigen-specific antibody was employed to modify nanogels because of the aberrant expression of Sialyl Tn (STn) antigen in pancreatic ductal adenocarcinoma (PDAC), resulting in the effective accumulation of drugs in tumor tissues, which not only improved the antitumor effect, but also implied a promising target for PDAC [71].

#### 3.6.4. Biomembrane Camouflaged

A protein corona is an unavoidable coating formed on the surface of nanoparticles during exposure to a biological milieu. Some studies have found that the targeting capacity of tumor-targeting ligands can be attenuated by the influence of the protein corona [72,73,74]. Meanwhile, some recent studies have also revealed that the targeting capacity of ligands can be partially retained after in vivo corona formation [75]. However, how the protein corona formed on the ligand-modified nanoparticle influences the targeting capacity remains unclear.

**Figure 7:**
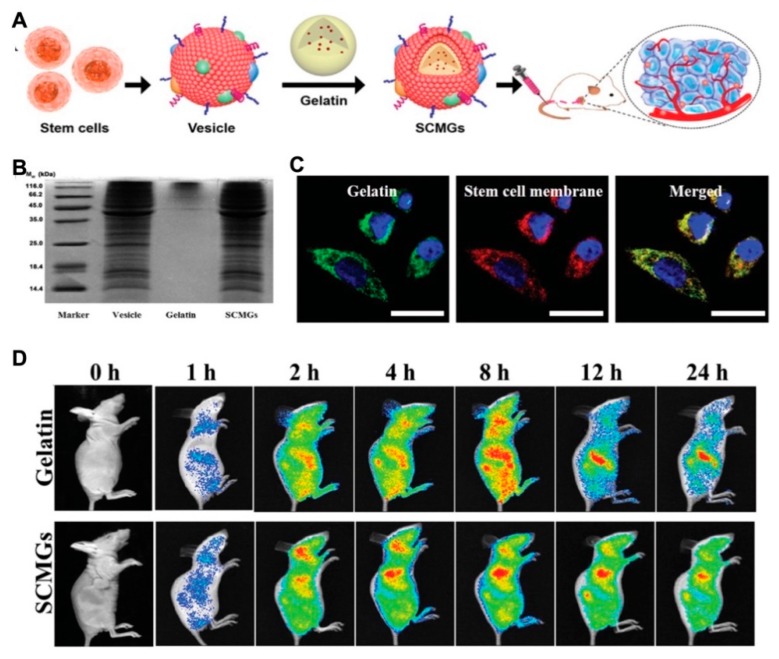
(**A**) The scheme illustrated the preparation of mesenchymal stem cell membrane-coated gelatin nanogels (SCMGs) and the tumor targeting property; (**B**) the surface protein analysis of SCMGs to certify the cell membrane coating; (**C**) confocal fluorescence images for the cell uptake of SCMGs which illustrated the colocalization of green labeled gelatin nanogels and red labeled stem cell membranes; (**D**) in vivo fluorescence imaging of mice after intravenous injection of SCMGs, that illustrated the enhanced tumor targeting and prolonged circulation after stem cell membrane coating. Reprinted with permission from (Small. 2016, 12(30), 4056–4062). Copyright (2016) Wiley Online Library.

A new strategy of biomembrane-camouflaged nanomedicine has been developed in recent studies for targeted delivery. Membrane-camouflaged DDSs can simulate the structure and function of cell membranes. Compared with conventional ligand-modified delivery systems, they displayed the superior passage through physiological barriers, more precise accumulation, prolonged circulation time and improved drug efficacy [76,77]. Mesenchymal stem cell membrane-coated gelatin nanogels (SCMGs) were loaded with DOX and exhibited considerable tumor-targeting ability because of tumor recognition by mesenchymal stem cells. Compared to the free DOX-treated group, the enhanced antitumor efficiency was observed in the group administrated by nanogels with mesenchymal stem cell coating, as well as the excellent biocompatibility with organs (Figure 7) [78].

## 4. Nanogels for Drug Delivery

Most nanogel systems are composed of synthetic polymers or natural biopolymers with crosslinked structures. The pores of the 3D network in nanogels are suitable for the incorporation of small molecules or biomacromolecules. Polymeric nanogels as drug carriers present the advantages of artificially controlling the dosage of drugs by external stimuli, shielding the irritating odor of drugs, improving the therapeutic efficacy and reducing the adverse effects of drugs [79]. Drugs with severe adverse effects, short circulation halftime, and easy degradability by enzymes, such as anticancer drugs, and proteins, are suitable for delivery by chemically crosslinked or physically assembled nanogel systems [80,81].

### 4.1. Small-Molecule Delivery

Nanogels display significant promise as DDSs due to their encapsulation stability and intelligent release, in addition to their water solubility, biocompatibility and biodegradability. The current study of nanogels is mainly focused on overcoming the instability of protein drugs to achieve targeted drug delivery to the tumor tissue.

DOX, as a hydrophilic model drug, is generally used to investigate the encapsulation and delivery properties of various nanogels [82]. The drug entrapping and controlled release could be easily fulfilled by swelling-collapse variation in the presence of environmental stimuli [83]. In addition to the hydrophilic molecules, some hydrophobic drugs could also be entrapped in the nanogel network. A Fe_3_O_4_ hybrid nanogel system was designed to load the hydrophobic drug rapamycin by interaction with the hydrophobic surface of Fe_3_O_4_ inner core. NIPMAm was introduced as the thermoresponsive monomer and 2-aminoethyl methacrylate hydrochloride (AEMA) as the pH-sensitive monomer to construct the hydrophilic shell of the hybrid nanogel. Furthermore, a collagen IV targeting peptide (KLWVLPK) was conjugated with the amino group of AEMA on the surface of drug-loaded nanogels, enabling the formulation of nanogels that targeted the injured artery, thereby achieving the vascular restenosis therapy [84].

### 4.2. Biomacromolecule Delivery

Unlike chemical drugs, biological macromolecules have the characteristics of large molecular weight, complex structure and biological functions; however, because of these characteristics, regulating the stability and permeability of biomacromolecules is challenging [85]. Therefore, such drugs are transported by a variety of nanometer DDSs [86,87], among which nanogels, composed of nanometer hydrogels, have an excellent drug-loading capacity, stability and hydrophilicity and the potential to be good carriers [88].

#### 4.2.1. Proteins Delivery

As proteins are characterized by poor stability, low permeability, enzymatic degradation and a short half-life, they need to be pharmaceutically modified for therapeutic purposes. Encapsulation in a variety of polymers is an effective way to control drug release and extend drug retention time [89]. Natural (e.g., chitosan, dextran, alginate) and synthetic polymers (e.g., polycaprolactone, acrylic polymers, polyallylamine) were introduced to form nanogels to deliver insulin, with both showing excellent biocompatibility, high permeability and enhanced glucose-based responsiveness. The remarkable advantages of these oral nanogels have enabled great effects on hypoglycemia. Despite the presence of epithelial barriers in the gastrointestinal tract, improved patient compliance was observed with oral insulin administration compared to insulin injection [90]. Mudassir et al. recently designed a pH-sensitive polymethyl methacrylate (MMA)/itaconic acid (IA) nanogel as a carrier for oral insulin to improve its bioavailability [91].

#### 4.2.2. Nucleic Acid Delivery

**Figure 8:**
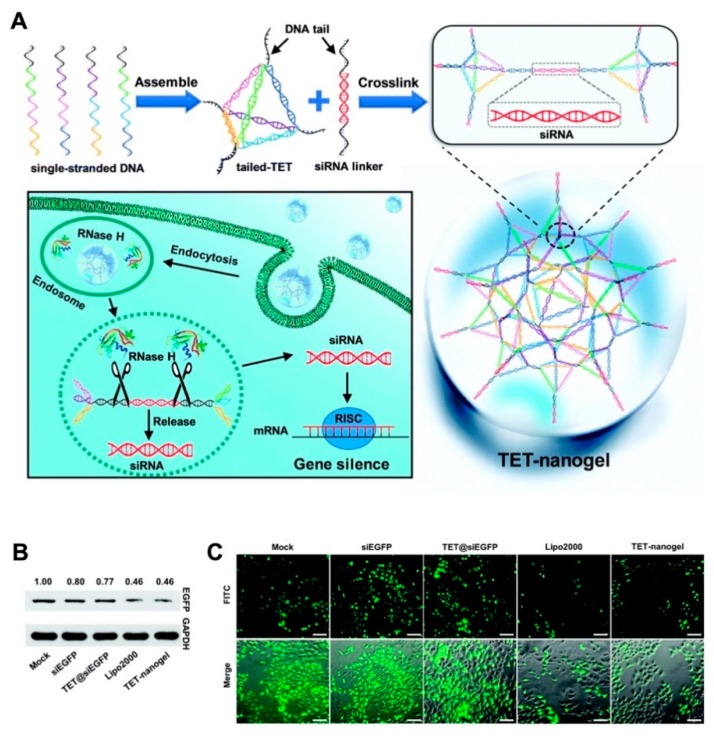
(**A**) The scheme for preparation of TET-nanogel and mechanism for the intracellular delivery of siRNA by crosslinking strategy; (**B**) Western blot analysis of EGFP expression by different vectors; (**C**) fluorescence microscopy images of gene silencing levels. Republished with permission of Royal Society of Chemistry, from [Chemical Communications. 2019, 55, 4222–4225. Edition Number 95, Copyright (2020)]; permission conveyed through Copyright Clearance Center, Inc.

Gene therapy is involved in the specific treatments of some genetic diseases by delivery of the therapeutic DNA or RNA sequences. Among these gene therapies, the application of small interfering RNA (siRNA) has become an important treatment for gene-related diseases due to the powerful ability of siRNAs to silence genes and effectively and specifically inhibit gene expression [92]. However, as nucleic acids, siRNAs are negatively charged hydrophilic compounds that cannot penetrate the cell surface, and their application is limited by some factors, such as their low transfection rates and short half-lives due to rapid enzymatic degradation [93]. To counter these problems, naked siRNA can be embellished by the biomolecule cholesterol, loaded in liposomes, or linked with polymer nanoparticles during nucleic acid treatment [94]. Furthermore, embellished siRNA can reach specific tissues because of its physicochemical characteristic. The siRNA therapy was realized by a nanogel-based delivery platform. Moreover, this approach is a clear direction for other functional oligonucleotide therapies. The tetrahedral DNA-based (TET) nanogel was introduced as a nonviral vector for siRNA assembly and provided protection during delivery. This strategy can prevent ribonuclease degradation and enable cell transfection effectively in vitro and in vivo, suggesting a promising platform for incorporating multiple devices for increased efficiency (Figure 8) [95].

## 5. Application of Nanogels in Combinational Therapy

Although different kinds of drugs were successfully encapsulated by nanogels in the abovementioned studies, the therapeutic outcomes were still undesirable, which can be largely ascribed to disadvantages including poor chemotherapeutic drug selectivity with the generation of side effects and development of drug resistance [96,97]. Moreover, the effect of cancer treatment with a single anticancer agent may be inconspicuous due to the complex nature of cancer occurrence and development [98]. Therefore, combination therapies, especially nanocarrier-based codelivery systems including micelles [99], liposomes [100], polymeric NPs [101], noble metal NPs [102] and inorganic NPs [103], have been thriving as promising strategies for the treatment of cancer. Nanogels are ideal candidates for drug codelivery due to their unique properties, such as their good biocompatibility, excellent stability, considerable loading capacity, as well as the controlled drug release ability with environmental stimulation [104].

### 5.1. Combinational Chemotherapy

**Figure 9:**
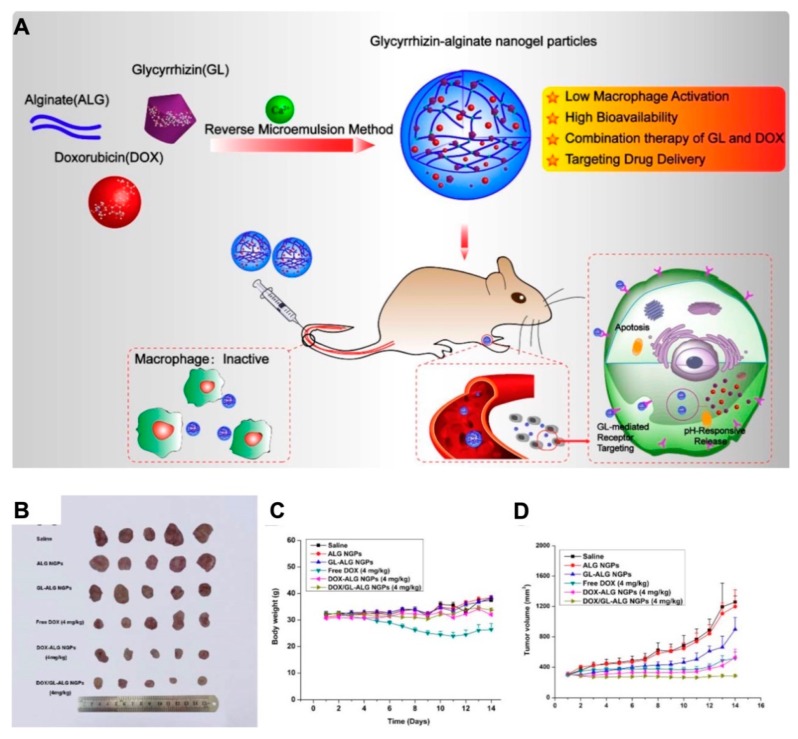
(**A**) The schematic illustration of alginate nanogel co-delivered with glycyrrhizin and doxorubicin (DOX/GL-ALG NGPs) for the improved anticancer therapy by inactivated macrophage and targeted drug delivery; the antitumor effect: photograph of tumors (**B**), body weight variation curve (**C**) and tumor growth curve (**D**) for each group after treatment. Reproduced from (Theranostics. 2019, 9, 6239–6255. Ivyspring International Publisher) distributed under the terms of Creative Commons Attribution 4.0 International License (CC-BY license).

Combinational chemotherapy is a route for the coadministration of multiple chemotherapeutic agents with enhanced therapeutic effects, which mostly utilize appropriate nanocarriers to deliver proportional drugs to the disease site [105]. Combinational therapy exhibits some advantages over routine single-drug chemotherapy. First, it can alleviate the toxicity and adverse effects of chemotherapeutic agents because the drug for combinational therapy is used at a lower dose than in single administration. Second, due to the different mechanisms of chemotherapeutic agents, distinct therapeutic targets would be activated simultaneously, reducing the development of drug resistance [106]. Additionally, the coadministration of agents by nanocarriers could display the on-demand drug release profiles to achieve therapeutic requirement. Despite the differences in distribution and metabolism of individual drugs, synergistic effects would prospectively be introduced by codelivery [97]. Nanogels provide a platform for drug codelivery, which is attributed to their 3D network structure for incorporating with both hydrophilic and hydrophobic compounds. In a recent study, both glycyrrhizin (GL) and DOX were encapsulated into alginate (ALG) nanogel particles via the phase inversion temperature emulsification method; the obtained drug-loaded alginate nanogels (DOX/GL-ALG NGPs) exhibited not only the hepatocellular carcinoma targeting property by GL, but also the synergistic antitumor effects of GL and DOX (Figure 9) [107]. In addition, ALG nanogels also presented favorable biocompatibility and low toxicity to hepatic tissues by controlled drug release [108]. These studies also implied that nanogels are an ideal nanoplatform for combination therapy.

### 5.2. Photo-Chemotherapy

Light has been used in the clinical setting since the beginning of the 20th century, and phototherapies have been widely used to treat skin diseases (such as lupus) and cancers [109,110]. Currently, phototherapies are divided into photodynamic therapy (PDT) and photothermal therapy (PTT). PTT uses photon energy to heat tumors physically, but PDT utilizes photosensitizers (PSs) to generate cytotoxic reactive oxygen species (ROS) and induce cancer cell death [111]. Phototherapies exhibit several advantages over traditional radiotherapy and chemotherapy, including their noninvasive nature, high selectivity, and a low number of side effects [112,113]. However, single phototherapy cannot completely eradicate a tumor because the laser intensity inevitably decays with the depth of the biological tissue, and the uneven distribution of the heat generated in the tissue damages normal tissues [114,115]. Given the nature of chemotherapy, a combination of phototherapy and chemotherapy based on nanogels as a DDS can effectively solve the above problems. Next, we will introduce the applications of photothermal chemotherapy and photodynamic chemotherapy.

#### 5.2.1. Photothermal Chemotherapy

**Figure 10:**
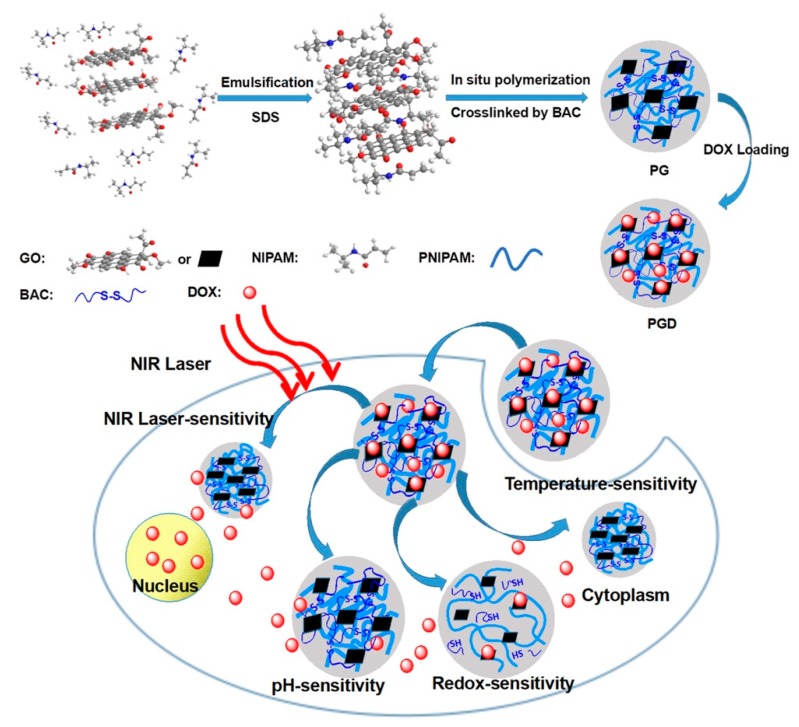
The schematic illustration of in situ polymerization of GO-hybridized nanogels (PG) and the DOX-loaded PG (PGD) with NIR laser-triggered and redox-responsive drug release, which exhibited the potentiality for combinational photothermal-chemotherapy. Adapted with permission from (ACS Appl. Bio Mater. 2018, 2, 330−338). Copyright (2018) American Chemical Society.

Recently, PTT, in which NIR light is absorbed, converted to heat via a photothermal agent and used to kill cancer cells, has emerged as a novel method to eliminate various types of cancer due to its simplicity, minimal nature and low systemic toxicity [116]. Most importantly, many studies have shown that the combination of PTT and chemotherapy can improve the efficiency of cancer therapy [117,118]. Hyperthermic conditions induced the efficient cell uptake of NPs with drug loading, stimulated drug release, and enhanced therapeutic outcomes. Moreover, chemo-photothermal combination therapy can augment cell membrane permeability [119,120]. Accordingly, many nanosystems for combined PTT and chemotherapy, including inorganic NPs [121], micelles [122], and polymeric NPs [123], have been developed. Nevertheless, a few critical issues lowered the therapeutic outcomes, such as uncontrolled release and low-dose drug accumulation in tumor tissue. The application of nanogels as an alternative option can moderately address the above issues [124]. In the recent study, the NIPAM monomer was crosslinked by reducible linker of BAC and in situ polymerized with graphene oxide (GO) to form the biodegradable hybridized nanogels (PG) with considerable biocompatibitlity. The redox-sensitivity of nanogels combined with the GO-induced thermal conversion assessed rapid drug release in tumor environment. This smart nanoplatform with local photothermal effect and controlled drug release exhibited the potential for chemo-photothermal synergistic therapy (Figure 10) [125].

#### 5.2.2. Photodynamic Chemotherapy

PDT has attracted widespread attention as a potential antitumor strategy by locally inducing cancer cell death [126]. PDT primarily involves the use of light at appropriate wavelengths to activate PSs, after which energy is transferred from activated PSs to molecular oxygen, producing highly toxic reactive oxygen species (ROS), especially the singlet oxygen (^1^O_2_), inducing tumor cell apoptosis and death [127,128]. Most of PDT processes are oxygen-dependent, and oxygen is continuously consumed during PDT, which severely limits the clinical application of PDT, especially in hypoxic solid tumors [129,130]. In addition, the limited penetration of tumor tissue by the laser reduces the efficacy of PDT [131]. Therefore, the combination of PDT and chemotherapy offers a potent approach to achieve synergistic anticancer effects [132,133]. Nanogels have been employed as a multifunctional delivery platform for chemo-photodynamic combinational therapy. For instance, a redox-sensitive nanogel was constructed for the delivery of the NHS-BODIPY-Br molecule, a heavy atom-modified reactive photosensitizer, and anticancer drug DOX. By the accumulation and controlled drug release in the tumor region, DOX loaded in the nanogel system induced the production of H_2_O_2_. Meanwhile, following laser irradiation, NHS-BODIPY-Br effectively converted H_2_O_2_ to ^1^O_2_, which resulted in the remarkably reduced cancer cell survival. The therapeutic effect implied that the nanogel system is a potential platform for combination chemotherapy with PDT [134].

### 5.3. Combinatorial Chemo-Immunotherapy

**Figure 11:**
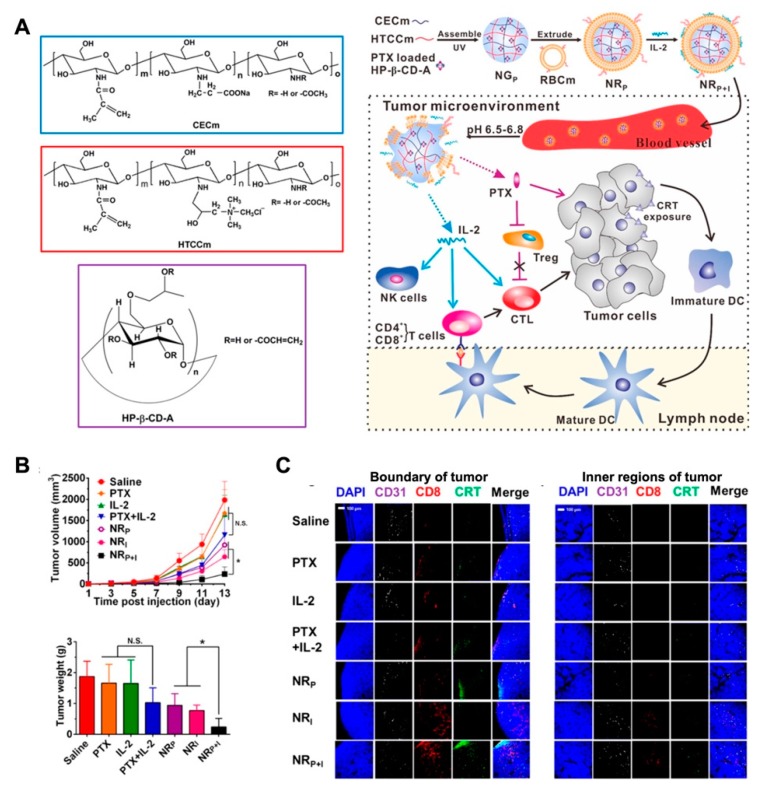
(**A**) The schematic illustration of the procedure of multifunctional nanogel NR_P+I_, and the mechanism for combinatorial chemo-immunotherapy; (**B**) the tumor volume and tumor weight of each group with corresponding treatment, which indicated the improved synergistic effect of chemo-immunotherapy; (**C**) immunofluorescence staining for of CD31, CRT and CD8+ cells in tumor sections of different treatments, which implied the remodulation of tumor microenvironment for combinatorial immunotherapy. Adapted with permission from (Nano Letters 2017, 17, (10), 6366–6375). Copyright (2017) American Chemical Society.

Polysaccharide-based self-assembly nanogels have been applied in tumor-associated antigen delivery in recent decades [135]. Daisuke M. et al. reported that the cholesteryl pullulan (CHP) nanogel could deliver a long peptide antigen (LPA) to the draining lymph node. By provoking a CD8+ T cell response through medullary macrophages, the CHP-based vaccine was proved the promising immunotherapeutic effect in animal models [136]. Unlike the directed induction of apoptosis or autophagy by the cytotoxic drugs, vaccine adjuvants or immunomodulators typically trigger antitumor immunity by regulating the tumor microenvironment [137,138,139]. However, the limitations of tumor immunotherapy include the high cost of therapy, the difficulty of sustained systematic immunity, and low patient response. The application of nanomedicine combined with immunotherapy is considered to be a promising, safe, and efficacious tumor treatment strategy [140]. In this study, the modified cyclodextrin was combined with chitosan derivates to form the pH-sensitive nanogels for paclitaxel (PTX) loading. Then, the PTX-load NG was further coated by an erythrocyte membrane with interleukin-2 (IL-2) binding to construct the NR_P+I_ nanoplatform for combinatorial immunotherapy. With the prolonged circulation and responsive drug release properties, the antitumor performance of NR_P+I_ in the melanoma model was superior to that of free drugs or monotreatment. With co-delivery of PTX and IL-2 to the tumor region, the low-dosage of PTX release triggered the calreticulin (CRT) exposure on tumor cells and further induced the infiltration of CD8+ T cell into tumor tissue. Furthermore, IL-2 cytokine stripped from the membrane of NR_P+I_ led to the activation of intratumoral cytotoxic T cells (CTLs) in the tumor microenvironment, which resulted in the synergistic effect of chemo-immunotherapy (Figure 11) [141].

## 6. Conclusions

Recently, nanogel-based nanoplatforms have become a tremendously promising system of drug delivery. In this work, the preparation, characterization, properties and biomedical applications of nanogels are reviewed in detail. Nanogels constructed by chemical crosslinking or physical self-assembly exhibit the ability to encapsulate hydrophilic or hydrophobic therapeutics, including but not limited to small-molecule compounds, proteins, DNA/RNA sequences, and even ultrasmall nanoparticles. The nanosized nature of the carriers endows them with a specific surface area and inner space, increasing the stability of loaded drugs and prolonging their circulation time in biological systems. Reactions or the cleavage of chemical bonds in the structure of nanogels have been shown to trigger the controlled or sustained drug release. Through the design of specific chemical structures and different methods of production, nanogels can realize diverse responsiveness (temperature-sensitive, pH-sensitive and redox-sensitive) and enable the stimuli-responsive release of drugs in the microenvironments of various diseases. To improve therapeutic outcomes and increase the precision of therapy, nanogels can be modified by specific ligands to achieve active targeting. The targeting ligand modification of nanogels, such as FA, cRGD, and STn antigen, can further enhance drug accumulation in disease sites. Moreover, biomembrane-camouflaged nanogels exhibit additional intelligent targeted delivery features. Consequently, the targeted delivery of therapeutic agents, as well as the combinational therapy strategy, result in the improved efficacy for disease treatments, by the introduction of a multifunctional nanogel-based DDS.

However, residual surfactants or unreacted monomers remaining in the nanogel formulation after synthesis are harmful substances with adverse effects in biomedicine. Physically linked nanogels might be more appropriate for drug formulation; however, the noncovalent bonds of physically linked nanogels are unstable, which might lead to premature leakage during circulation. Meanwhile, as a drug carrier, the drug-loading capacity and controlled drug release behavior of nanogels need to be further improved, especially for the release of certain drugs at large doses with continuous administration. In addition, the biosafety and in vivo degradation of nanogels still need exhaustive investigation because the components of nanogels are artificial polymeric materials

## References

[1] Cui W., Li J., Decher G. (2016). Self-assembled smart nanocarriers for targeted drug delivery. Adv. Mater..

[2] Torchilin V.P. (2014). Multifunctional, stimuli-sensitive nanoparticulate systems for drug delivery. Nat. Rev. Drug Discov..

[3] Jiang Y., Chen J., Deng C., Suuronen E.J., Zhong Z. (2014). Click hydrogels, microgels and nanogels: Emerging platforms for drug delivery and tissue engineering. Biomaterials.

[4] Soni K.S., Desale S.S., Bronich T.K. (2016). Nanogels: An overview of properties, biomedical applications and obstacles to clinical translation. J. Control Release.

[5] Li Y., Maciel D., Rodrigues J., Shi X., Tomás H. (2015). Biodegradable polymer nanogels for drug/nucleic acid delivery. Chem. Rev..

[6] Raemdonck K., Demeester J., De Smedt S. (2009). Advanced nanogel engineering for drug delivery. Soft Matter.

[7] Sahiner N., Godbey W.T., McPherson G., John V. (2006). Microgel, nanogel and hydrogel-hydrogel semi-IPN composites for biomedical applications: Synthesis and characterization. Colloid Polym. Sci..

[8] Vinogradov S.V., Bronich T.K., Kabanov A.V. (2002). Nanosized cationic hydrogels for drug delivery: Preparation, properties and interactions with cells. Adv. Drug Deliv. Rev..

[9] Maya S., Bruno S., Amrita N., Rejinold N.S., Shantikumar V.N., Jayakumar R. (2013). Smart stimuli sensitive nanogels in cancer drug delivery and imaging: A review. Curr. Pharm. Des..

[10] Ashrafizadeh M., Tam K.C., Javadi A., Abdollahi M., Sadeghnejad S., Bahramian A. (2019). Synthesis and physicochemical properties of dual-responsive acrylic acid/butyl acrylate cross-linked nanogel systems. J. Colloid Interface Sci..

[11] Wang H., Chen Q., Zhou S. (2018). Carbon-based hybrid nanogels: A synergistic nanoplatform for combined biosensing, bioimaging, and responsive drug delivery. Chem. Soc. Rev..

[12] Theune L.E., Buchmann J., Wedepohl S., Molina M., Laufer J., Calderón M. (2019). NIR- and thermo-responsive semi-interpenetrated polypyrrole nanogels for imaging guided combinational photothermal and chemotherapy. J. Control Release.

[13] Kabanov A.V., Vinogradov S.V. (2009). Nanogels as pharmaceutical carriers: Finite networks of infinite capabilities. Angew. Chem.Int. Edit..

[14] Szilágyi B.Á., Némethy Á., Magyar A., Szabó I., Bősze S., Gyarmati B., Szilágyi A. (2018). Amino acid based polymer hydrogel with enzymatically degradable cross-links. React. Funct. Polym..

[15] Peres L.B., dos Anjos R.S., Tappertzhofen L.C., Feuser P.E., de Araújo P.H.H., Landfester K., Sayer C., Muñoz-Espí R. (2018). pH-responsive physically and chemically cross-linked glutamic-acid-based hydrogels and nanogels. Eur. Polym. J..

[16] Cortez-Lemus N.A., Licea-Claverie A. (2016). Poly (N-vinylcaprolactam), a comprehensive review on a thermoresponsive polymer becoming popular. Prog. Polym. Sci..

[17] Etchenausia L., Khoukh A., Deniau Lejeune E., Save M. (2017). RAFT/MADIX emulsion copolymerization of vinyl acetate and N-vinylcaprolactam: Towards waterborne physically crosslinked thermoresponsive particles. Polym. Chem..

[18] Buwalda S.J., Vermonden T., Hennink W.E. (2017). Hydrogels for therapeutic delivery: Current developments and future directions. Biomacromolecules.

[19] Wang J., Wang X., Yan G., Fu S., Tang R. (2017). pH-sensitive nanogels with ortho ester linkages prepared via thiol-ene click chemistry for efficient intracellular drug release. J. Colloid Interface Sci..

[20] Dispenza C., Spadaro G., Jonsson M. (2016). Radiation engineering of multifunctional nanogels. Top. Curr. Chem..

[21] He J., Tong X., Zhao Y. (2009). Photoresponsive nanogels based on photocontrollable cross-links. Macromolecules.

[22] Denmark D.J., Hyde R.H., Gladney C., Phan M.-H., Bisht K.S., Srikanth H., Mukherjee P., Witanachchi S. (2017). Photopolymerization-based synthesis of iron oxide nanoparticle embedded PNIPAM nanogels for biomedical applications. Drug Deliv..

[23] Murphy E.A., Majeti B.K., Mukthavaram R., Acevedo L.M., Barnes L.A., Cheresh D.A. (2011). Targeted nanogels: A versatile platform for drug delivery to tumors. Mol. Cancer Ther..

[24] Bhardwaj A., Kumar L., Mehta S., Mehta A. (2015). Stimuli-sensitive systems-an emerging delivery system for drugs. Artif. Cells Nanomed. Biotechnol..

[25] Yu J., Chu X., Hou Y. (2014). Stimuli-responsive cancer therapy based on nanoparticles. Chem. Commun..

[26] Smeets N.M.B., Hoare T. (2013). Designing responsive microgels for drug delivery applications. J. Polym. Sci. Pol. Chem..

[27] Lee H., Fonge H., Hoang B., Reilly R.M., Allen C. (2010). The effects of particle size and molecular targeting on the intratumoral and subcellular distribution of polymeric nanoparticles. Mol. Pharm..

[28] Liu R., Hu C., Yang Y., Zhang J., Gao H. (2019). Theranostic nanoparticles with tumor-specific enzyme-triggered size reduction and drug release to perform photothermal therapy for breast cancer treatment. Acta Pharm. Sin. B.

[29] Liu R., Xiao W., Hu C., Xie R., Gao H. (2018). Theranostic size-reducible and no donor conjugated gold nanocluster fabricated hyaluronic acid nanoparticle with optimal size for combinational treatment of breast cancer and lung metastasis. J. Control Release.

[30] Qian J., Wu F. (2013). Thermosensitive PNIPAM semi-hollow spheres for controlled drug release. J. Mat. Chem. B.

[31] Wang D., Huang H., Zhou M., Lu H., Chen J., Chang Y.-T., Gao J., Chai Z., Hu Y. (2019). A thermoresponsive nanocarrier for mitochondria-targeted drug delivery. Chem. Commun..

[32] Tokuyama H., Kato Y. (2008). Preparation of poly (N-isopropylacrylamide) emulsion gels and their drug release behaviors. Colloid Surf. B Biointerfaces.

[33] Chen Y., Ballard N., Bon S. (2013). Moldable high internal phase emulsion hydrogel objects from non-covalently crosslinked poly(N-isopropylacrylamide) nanogel dispersions. Chem. Commun..

[34] Wang G., Xie R., Ju X.J., Chu L.Y. (2012). Thermo-responsive polyethersulfone composite membranes blended with poly(N-isopropylacrylamide) nanogels. Chem. Eng. Technol..

[35] Ashraf S., Park H.-K., Park H., Lee S.-H. (2016). Snapshot of phase transition in thermoresponsive hydrogel PNIPAM: Role in drug delivery and tissue engineering. Macromol. Res..

[36] Gan J., Guan X., Zheng J., Guo H., Wu K., Liang L., Lu M. (2016). Biodegradable, thermoresponsive PNIPAM-based hydrogel scaffolds for the sustained release of levofloxacin. RSC Adv..

[37] Cao M., Wang Y., Hu X., Gong H., Li R., Cox H., Zhang J., Waigh T.A., Xu H., Lu J.R. (2019). Reversible thermoresponsive peptide–PNIPAM hydrogels for controlled drug delivery. Biomacromolecules.

[38] Pan G., Guo Q., Cao C., Yang H., Li B. (2013). Thermo-responsive molecularly imprinted nanogels for specific recognition and controlled release of proteins. Soft Matter.

[39] Chen W., Meng F., Li F., Ji S.-J., Zhong Z. (2009). pH-Responsive biodegradable micelles based on acid-labile polycarbonate hydrophobe: Synthesis and triggered drug release. Biomacromolecules.

[40] Du J., Tang Y., Lewis A.L., Armes S.P. (2005). pH-sensitive vesicles based on a biocompatible zwitterionic diblock copolymer. J. Am. Chem. Soc..

[41] Cheng R., Meng F., Deng C., Klok H.-A., Zhong Z. (2013). Dual and multi-stimuli responsive polymeric nanoparticles for programmed site-specific drug delivery. Biomaterials.

[42] Argentiere S., Blasi L., Morello G., Gigli G. (2011). A novel pH-responsive nanogel for the controlled uptake and release of hydrophobic and cationic solutes. J. Phys. Chem. C.

[43] Chen Z., Wu C., Zhang Z., Wu W., Wang X., Yu Z. (2018). Synthesis, functionalization, and nanomedical applications of functional magnetic nanoparticles. Chin. Chem. Lett..

[44] Pan J., Hu P., Guo Y., Hao J., Ni D., Xu Y., Bao Q., Yao H., Wei C., Wu Q. (2020). Combined magnetic hyperthermia and immune therapy for primary and metastatic tumor treatments. ACS Nano.

[45] Cazares-Cortes E., Espinosa A., Guigner J.M., Michel A., Griffete N., Wilhelm C., Ménager C. (2017). Doxorubicin intracellular remote release from biocompatible oligo (ethylene glycol) methyl ether methacrylate-based magnetic nanogels triggered by magnetic hyperthermia. ACS Appl. Mater. Interfaces..

[46] Seah B.C., Teo B.M. (2018). Recent advances in ultrasound-based transdermal drug delivery. Int. J. Nanomed..

[47] Fan C.H., Lin C.Y., Liu H.L., Yeh C.K. (2017). Ultrasound targeted CNS gene delivery for Parkinson’s disease treatment. J. Control Release.

[48] Chen M., Liang X., Gao C., Zhao R., Zhang N., Wang S., Chen W., Zhao B., Wang J., Dai Z. (2018). Ultrasound triggered conversion of porphyrin/camptothecin-fluoroxyuridine triad microbubbles into nanoparticles overcomes multidrug resistance in colorectal cancer. ACS Nano.

[49] Qiao L., Wang X., Gao Y., Wei Q., Hu W., Wu L., Li P., Zhu R., Wang Q. (2016). Laccase-mediated formation of mesoporous silica nanoparticle based redox stimuli-responsive hybrid nanogels as a multifunctional nanotheranostic agent. Nanoscale.

[50] Gao W., Hu Y., Xu L., Liu M., He B. (2018). Dual pH and glucose sensitive gel gated mesoporous silica nanoparticles for drug delivery. Chin. Chem. Lett..

[51] Samah N.H.A., Heard C.M. (2013). Enhanced in vitro transdermal delivery of caffeine using a temperature- and pH-sensitive nanogel, poly (NIPAM-co-AAc). Int. J. Pharm..

[52] Nita L.E., Chiriac A.P., Diaconu A., Tudorachi N., Mititelu-Tartau L. (2016). Multifunctional nanogels with dual temperature and pH responsiveness. Int. J. Pharm..

[53] Morimoto N., Qiu X.-P., Winnik F.M., Akiyoshi K. (2008). Dual stimuli-responsive nanogels by self-assembly of polysaccharides lightly grafted with thiol-terminated poly (N-isopropylacrylamide) chains. Macromolecules.

[54] Peng J., Qi T., Liao J., Fan M., Luo F., Li H., Qian Z. (2012). Synthesis and characterization of novel dual-responsive nanogels and their application as drug delivery systems. Nanoscale.

[55] Peng J., Qi T., Liao J., Chu B., Yang Q., Li W., Qu Y., Luo F., Qian Z. (2013). Controlled release of cisplatin from pH-thermal dual responsive nanogels. Biomaterials.

[56] Cheng R., Feng F., Meng F., Deng C., Feijen J., Zhong Z. (2011). Glutathione-responsive nano-vehicles as a promising platform for targeted intracellular drug and gene delivery. J. Control Release.

[57] Zhan Y., Gonçalves M., Yi P., Capelo D., Zhang Y., Rodrigues J., Liu C., Tomás H., Li Y., He P. (2015). Thermo/redox/pH-triple sensitive poly (N-isopropylacrylamide-co-acrylic acid) nanogels for anticancer drug delivery. J. Mat. Chem. B.

[58] Luo Z., Dai Y., Gao H. (2019). Development and application of hyaluronic acid in tumor targeting drug delivery. Acta Pharm. Sin. B.

[59] Yoo J., Park C., Yi G., Lee D., Koo H. (2019). Active targeting strategies using biological ligands for nanoparticle drug delivery systems. Cancers.

[60] Low P.S., Kularatne S.A. (2009). Folate-targeted therapeutic and imaging agents for cancer. Curr. Opin. Chem. Biol..

[61] Nukolova N.V., Oberoi H.S., Cohen S.M., Kabanov A.V., Bronich T.K. (2011). Folate-decorated nanogels for targeted therapy of ovarian cancer. Biomaterials.

[62] Huang X., Yin Y., Wu M., Zan W., Yang Q. (2019). LyP-1 peptide-functionalized gold nanoprisms for SERRS imaging and tumor growth suppressing by PTT induced-hyperthermia. Chin. Chem. Lett..

[63] Peng J., Yang Q., Xiao Y., Shi K., Liu Q., Hao Y., Yang F., Han R., Qian Z. (2019). Tumor microenvironment responsive drug-dye-peptide nanoassembly for enhanced tumor-targeting, penetration, and photo-chemo-immunotherapy. Adv. Funct. Mater..

[64] Yang Q., Peng J., Shi K., Xiao Y., Liu Q., Han R., Wei X., Qian Z. (2019). Rationally designed peptide-conjugated gold/platinum nanosystem with active tumor-targeting for enhancing tumor photothermal-immunotherapy. J. Control Release.

[65] Ruoslahti E., Pierschbacher M.D. (1986). Arg-Gly-Asp: A versatile cell recognition signal. Cell.

[66] Gao X., Yang H., Wu M., Shi K., Zhou C., Peng J., Yang Q. (2018). Targeting delivery of lidocaine and cisplatin by nanogel enhances chemotherapy and alleviates metastasis. ACS Appl. Mater. Interfaces.

[67] Nukolova N.V., Yang Z., Kim J.O., Kabanov A.V., Bronich T.K. (2011). Polyelectrolyte nanogels decorated with monoclonal antibody for targeted drug delivery. React. Funct. Polym..

[68] Lewis Phillips G.D., Li G., Dugger D.L., Crocker L.M., Parsons K.L., Mai E., Blättler W.A., Lambert J.M., Chari R.V.J., Lutz R.J. (2008). Targeting HER2-positive breast cancer with trastuzumab-DM1, an antibody–cytotoxic drug conjugate. Cancer Res..

[69] Cheng W.W., Allen T.M. (2010). The use of single chain Fv as targeting agents for immunoliposomes: An update on immunoliposomal drugs for cancer treatment. Expert Opin. Drug Deliv..

[70] Glennie M.J., van de Winkel J.G.J. (2003). Renaissance of cancer therapeutic antibodies. Drug Discov. Today.

[71] Soni K.S., Thomas D., Caffrey T., Mehla K., Lei F., Connell K.A., Sagar S., Lele S.M., Hollingsworth M.A., Radhakrishnan P. (2019). A polymeric nanogel-based treatment regimen for enhanced efficacy and sequential administration of synergistic drug combination in pancreatic cancer. J. Pharmacol. Exp. Ther..

[72] Salvati A., Pitek A., Monopoli M., Prapainop K., Bombelli F., Hristov D., Kelly P., Aberg C., Mahon E., Dawson K. (2013). Transferrin-functionalized nanoparticles lose their targeting capabilities when a biomolecule corona adsorbs on the surface. Nat. Nanotechnol..

[73] Xiao W., Xiong J., Zhang S., Xiong Y., Zhang H., Gao H. (2018). Influence of ligands property and particle size of gold nanoparticles on the protein adsorption and corresponding targeting ability. Int. J. Pharm..

[74] Gao H., He Q. (2014). The interaction of nanoparticles with plasma proteins and the consequent influence on nanoparticles behavior. Expert Opin. Drug Deliv..

[75] Zhang H., Wu T., Yu W., Ruan S., He Q., Gao H. (2018). Ligand size and conformation affect the behavior of nanoparticles coated with in vitro and in vivo protein corona. ACS Appl. Mater. Interfaces.

[76] Fang R.H., Jiang Y., Fang J.C., Zhang L. (2017). Cell membrane-derived nanomaterials for biomedical applications. Biomaterials.

[77] Yang Q., Xiao Y., Yin Y., Li G., Peng J. (2019). Erythrocyte membrane-camouflaged IR780 and DTX coloading polymeric nanoparticles for imaging-guided cancer Photo–Chemo combination therapy. Mol. Pharm..

[78] Gao C., Lin Z., Jurado-Sánchez B., Lin X., Wu Z., He Q. (2016). Stem cell membrane-coated nanogels for highly efficient in vivo tumor targeted drug delivery. Small.

[79] Chacko R.T., Ventura J., Zhuang J., Thayumanavan S. (2012). Polymer nanogels: A versatile nanoscopic drug delivery platform. Adv. Drug Deliv. Rev..

[80] Grimaudo M.A., Concheiro A., Alvarez-Lorenzo C. (2019). Nanogels for regenerative medicine. J. Control Release.

[81] Cuggino J.C., Blanco E.R.O., Gugliotta L.M., Alvarez Igarzabal C.I., Calderón M. (2019). Crossing biological barriers with nanogels to improve drug delivery performance. J. Control Release.

[82] Xu L., Su T., Xu X., Zhu L., Shi L. (2019). Platelets membrane camouflaged irinotecan-loaded gelatin nanogels for in vivo colorectal carcinoma therapy. J. Drug Deliv. Sci. Technol..

[83] Hajebi S., Rabiee N., Bagherzadeh M., Ahmadi S., Rabiee M., Roghani-Mamaqani H., Tahriri M., Tayebi L., Hamblin M.R. (2019). Stimulus-responsive polymeric nanogels as smart drug delivery systems. Acta Biomater..

[84] Yang Q., Peng J., Chen C., Xiao Y., Tan L., Xie X., Xu X., Qian Z. (2018). Targeting delivery of rapamycin with anti-collagen IV peptide conjugated Fe_3_O_4_@nanogels system for vascular restenosis therapy. J. Biomed. Nanotechnol..

[85] Li D., van Nostrum C.F., Mastrobattista E., Vermonden T., Hennink W.E. (2017). Nanogels for intracellular delivery of biotherapeutics. J. Control Release.

[86] Nosrati H., Abbasi R., Charmi J., Rakhshbahar A., Aliakbarzadeh F., Danafar H., Davaran S. (2018). Folic acid conjugated bovine serum albumin: An efficient smart and tumor targeted biomacromolecule for inhibition folate receptor positive cancer cells. Int. J. Biol. Macromol..

[87] Zhang H., Zhai Y., Wang J., Zhai G. (2016). New progress and prospects: The application of nanogel in drug delivery. Mater. Sci. Eng. C.

[88] Wu H.-Q., Wang C.-C. (2016). Biodegradable smart nanogels: A new platform for targeting drug delivery and biomedical diagnostics. Langmuir.

[89] Ye C., Chi H. (2018). A review of recent progress in drug and protein encapsulation: Approaches, applications and challenges. Mater. Sci. Eng. C.

[90] Fonte P., Araújo F., Silva C., Pereira C., Reis S., Santos H.A., Sarmento B. (2015). Polymer-based nanoparticles for oral insulin delivery: Revisited approaches. Biotechnol. Adv..

[91] Mudassir J., Darwis Y., Muhamad S., Khan A.A. (2019). Self-assembled insulin and nanogels polyelectrolyte complex (Ins/NGs-PEC) for oral insulin delivery: Characterization, lyophilization and in-vivo evaluation. Int. J. Nanomed..

[92] Itani R., Al Faraj A. (2019). siRNA conjugated nanoparticles-A next generation strategy to treat lung cancer. Int. J. Mol. Sci..

[93] Vicentini F.T.M.d.C., Borgheti-Cardoso L.N., Depieri L.V., de Macedo Mano D., Abelha T.F., Petrilli R., Bentley M.V.L.B. (2013). Delivery systems and local administration routes for therapeutic siRNA. Pharm. Res..

[94] Ni R., Feng R., Chau Y. (2019). Synthetic approaches for nucleic acid delivery: Choosing the right carriers. Life.

[95] Xue H., Ding F., Zhang J., Guo Y., Gao X., Feng J., Zhu X., Zhang C. (2019). DNA tetrahedron-based nanogels for siRNA delivery and gene silencing. Chem. Commun..

[96] Qi S.-S., Sun J.-H., Yu H.-H., Yu S.-Q. (2017). Co-delivery nanoparticles of anti-cancer drugs for improving chemotherapy efficacy. Drug Deliv..

[97] Reza Baradaran E., Niloufar M., Shabnam S., Ali Z., Farid Abedin D. (2019). Co-delivery nanosystems for cancer treatment: A review. Pharm. Nanotechnol..

[98] Jhaveri A., Deshpande P., Torchilin V. (2014). Stimuli-sensitive nanopreparations for combination cancer therapy. J. Control Release.

[99] Ghamkhari A., Pouyafar A., Salehi R., Rahbarghazi R. (2019). Chrysin and docetaxel loaded biodegradable micelle for combination chemotherapy of cancer stem cell. Pharm. Res..

[100] Fu J., Li W., Xin X., Chen D., Hu H. (2019). Transferrin modified nano-liposome co-delivery strategies for enhancing the cancer therapy. J. Pharm. Sci..

[101] Saneja A., Kumar R., Mintoo M.J., Dubey R.D., Sangwan P.L., Mondhe D.M., Panda A.K., Gupta P.N. (2019). Gemcitabine and betulinic acid co-encapsulated PLGA−PEG polymer nanoparticles for improved efficacy of cancer chemotherapy. Mater. Sci. Eng. C.

[102] Fan L., Yang Q., Tan J., Qiao Y., Wang Q., He J., Wu H., Zhang Y. (2015). Dual loading miR-218 mimics and Temozolomide using AuCOOH@FA-CS drug delivery system: Promising targeted anti-tumor drug delivery system with sequential release functions. J. Exp. Clin. Cancer Res..

[103] Wang F., Zhang L., Bai X., Cao X., Jiao X., Huang Y., Li Y., Qin Y., Wen Y. (2018). Stimuli-responsive nano-carrier for co-delivery of MiR-31 and doxorubicin to suppress high MtEF4 cancer. ACS Appl. Mater. Interfaces.

[104] Vashist A., Kaushik A., Vashist A., Bala J., Nikkhah-Moshaie R., Sagar V., Nair M. (2018). Nanogels as potential drug nanocarriers for CNS drug delivery. Drug Discov. Today.

[105] Lehár J., Krueger A.S., Avery W., Heilbut A.M., Johansen L.M., Price E.R., Rickles R.J., Short Iii G.F., Staunton J.E., Jin X. (2009). Synergistic drug combinations tend to improve therapeutically relevant selectivity. Nat. Biotechnol..

[106] He C., Tang Z., Tian H., Chen X. (2016). Co-delivery of chemotherapeutics and proteins for synergistic therapy. Adv. Drug Deliv. Rev..

[107] Wang Q.-S., Gao L.-N., Zhu X.-N., Zhang Y., Zhang C.-N., Xu D., Cui Y.-L. (2019). Co-delivery of glycyrrhizin and doxorubicin by alginate nanogel particles attenuates the activation of macrophage and enhances the therapeutic efficacy for hepatocellular carcinoma. Theranostics.

[108] Tong X.-F., Zhao F.-Q., Ren Y.-Z., Zhang Y., Cui Y.-L., Wang Q.-S. (2018). Injectable hydrogels based on glycyrrhizin, alginate, and calcium for three-dimensional cell culture in liver tissue engineering. J. Biomed. Mater. Res. Part A.

[109] Li Y., Li X., Zhou F., Doughty A., Hoover A.R., Nordquist R.E., Chen W.R. (2019). Nanotechnology-based photoimmunological therapies for cancer. Cancer Lett..

[110] Grzybowski A., Pietrzak K. (2012). From patient to discoverer—Niels Ryberg Finsen (1860–1904)—the founder of phototherapy in dermatology. Clin. Dermatol..

[111] Zhu H., Cheng P., Chen P., Pu K. (2018). Recent progress in the development of near-infrared organic photothermal and photodynamic nanotherapeutics. Biomater. Sci..

[112] Cheng L., Wang C., Feng L., Yang K., Liu Z. (2014). Functional nanomaterials for phototherapies of cancer. Chem. Rev..

[113] Girma W.M., Tzing S.-H., Tseng P.-J., Huang C.-C., Ling Y.-C., Chang J.-Y. (2018). Synthesis of cisplatin (IV) prodrug-tethered CuFeS2 nanoparticles in tumor-targeted chemotherapy and photothermal therapy. ACS Appl. Mater. Interfaces.

[114] Luo D., Carter K.A., Miranda D., Lovell J.F. (2017). Chemophototherapy: An emerging treatment option for solid tumors. Adv. Sci..

[115] Bao T., Yin W., Zheng X., Zhang X., Yu J., Dong X., Yong Y., Gao F., Yan L., Gu Z. (2016). One-pot synthesis of PEGylated plasmonic MoO3–x hollow nanospheres for photoacoustic imaging guided chemo-photothermal combinational therapy of cancer. Biomaterials.

[116] Chen Q., Wen J., Li H., Xu Y., Liu F., Sun S. (2016). Recent advances in different modal imaging-guided photothermal therapy. Biomaterials.

[117] Khafaji M., Zamani M., Golizadeh M., Bavi O. (2019). Inorganic nanomaterials for chemo/photothermal therapy: A promising horizon on effective cancer treatment. Biophys. Rev..

[118] Ruttala H.B., Ramasamy T., Poudel B.K., Ruttala R.R.T., Jin S.G., Choi H.-G., Ku S.-K., Yong C.S., Kim J.O. (2020). Multi-responsive albumin-lonidamine conjugated hybridized gold nanoparticle as a combined photothermal-chemotherapy for synergistic tumor ablation. Acta Biomater..

[119] Cao J., Chen Z., Chi J., Sun Y., Sun Y. (2018). Recent progress in synergistic chemotherapy and phototherapy by targeted drug delivery systems for cancer treatment. Artif. Cell. Nanomed. Biotechnol..

[120] Li Z., Chen Y., Yang Y., Yu Y., Zhang Y., Zhu D., Yu X., Ouyang X., Xie Z., Zhao Y. (2019). Recent advances in nanomaterials-based chemo-photothermal combination therapy for improving cancer treatment. Front. Bioeng. Biotechnol..

[121] Wang M., Liang Y., Zhang Z., Ren G., Liu Y., Wu S., Shen J. (2019). Ag@Fe3O4@C nanoparticles for multi-modal imaging-guided chemo-photothermal synergistic targeting for cancer therapy. Anal. Chim. Acta.

[122] Li Z., Wang H., Chen Y., Wang Y., Li H., Han H., Chen T., Jin Q., Ji J. (2016). pH- and NIR light-responsive polymeric prodrug micelles for hyperthermia-assisted site-specific chemotherapy to reverse drug resistance in cancer treatment. Small.

[123] Yang Z., Wang J., Liu S., Li X., Miao L., Yang B., Zhang C., He J., Ai S., Guan W. (2020). Defeating relapsed and refractory malignancies through a nano-enabled mitochondria-mediated respiratory inhibition and damage pathway. Biomaterials.

[124] Qi Y., Min H., Mujeeb A., Zhang Y., Han X., Zhao X., Anderson G.J., Zhao Y., Nie G. (2018). Injectable hexapeptide hydrogel for localized chemotherapy prevents breast cancer recurrence. ACS Appl. Mater. Interfaces.

[125] Zhang W., Ai S., Ji P., Liu J., Li Y., Zhang Y., He P. (2018). Photothermally enhanced chemotherapy delivered by graphene oxide-based multiresponsive nanogels. ACS Appl. Bio. Mater..

[126] Sztandera K., Gorzkiewicz M., Klajnert-Maculewicz B. (2019). Nanocarriers in photodynamic therapy—in vitro and in vivo studies. Wiley Interdiscip Rev. Nanomed. Nanobiotechnol..

[127] Li X., Kwon N., Guo T., Liu Z., Yoon J. (2018). Innovative strategies for hypoxic-tumor photodynamic therapy. Angew. Chem.Int. Edit..

[128] Fan W., Huang P., Chen X. (2016). Overcoming the Achilles’ heel of photodynamic therapy. Chem. Soc. Rev..

[129] Chen H., Tian J., He W., Guo Z. (2015). H2O2-activatable and O2-evolving nanoparticles for highly efficient and selective photodynamic therapy against hypoxic tumor cells. J. Am. Chem. Soc..

[130] Luo Z., Li M., Zhou M., Li H., Chen Y., Ren X., Dai Y. (2019). O2-evolving and ROS-activable nanoparticles for treatment of multi-drug resistant cancer by combination of photodynamic therapy and chemotherapy. Nanomed. Nanotechnol. Biol. Med..

[131] Wu H., Zeng F., Zhang H., Xu J., Qiu J., Wu S. (2016). A nanosystem capable of releasing a photosensitizer bioprecursor under two-photon irradiation for photodynamic therapy. Adv. Sci..

[132] Tian G., Zhang X., Gu Z., Zhao Y. (2015). Recent advances in upconversion nanoparticles-based multifunctional nanocomposites for combined cancer therapy. Adv. Mater..

[133] Yi X., Dai J., Han Y., Xu M., Zhang X., Zhen S., Zhao Z., Lou X., Xia F. (2018). A high therapeutic efficacy of polymeric prodrug nano-assembly for a combination of photodynamic therapy and chemotherapy. Commun. Biol..

[134] Liu L., Li T., Ruan Z., Yuan P., Yan L. (2018). Reduction-sensitive polypeptide nanogel conjugated BODIPY-Br for NIR imaging-guided chem/photodynamic therapy at low light and drug dose. Mater. Sci. Eng. C.

[135] Tahara Y., Akiyoshi K. (2015). Current advances in self-assembled nanogel delivery systems for immunotherapy. Adv. Drug Deliv. Rev..

[136] Muraoka D., Harada N., Hayashi T., Tahara Y., Momose F., Sawada S.-I., Mukai S.-A., Akiyoshi K., Shiku H. (2014). Nanogel-based immunologically stealth vaccine targets macrophages in the medulla of lymph node and induces potent antitumor immunity. ACS Nano.

[137] Song C., Phuengkham H., Kim Y.S., Dinh V.V., Lee I., Shin I.W., Shin H.S., Jin S.M., Um S.H., Lee H. (2019). Syringeable immunotherapeutic nanogel reshapes tumor microenvironment and prevents tumor metastasis and recurrence. Nat. Commun..

[138] Nuhn L., Vanparijs N., De Beuckelaer A., Lybaert L., Verstraete G., Deswarte K., Lienenklaus S., Shukla N.M., Salyer A.C.D., Lambrecht B.N. (2016). pH-degradable imidazoquinoline-ligated nanogels for lymph node-focused immune activation. Proc. Natl. Acad. Sci. USA.

[139] Purwada A., Tian Y.F., Huang W., Rohrbach K.M., Deol S., August A., Singh A. (2016). Self-assembly protein nanogels for safer cancer immunotherapy. Adv. Healthc. Mater..

[140] Nam J., Son S., Park K.S., Zou W., Shea L.D., Moon J.J. (2019). Cancer nanomedicine for combination cancer immunotherapy. Nat. Rev. Mater..

[141] Song Q., Yin Y., Shang L., Wu T., Zhang D., Kong M., Zhao Y., He Y., Tan S., Guo Y. (2017). Tumor microenvironment responsive nanogel for the combinatorial antitumor effect of chemotherapy and immunotherapy. Nano Lett..

